# Kinetics of vaccine-induced neutralizing antibody titers and estimated protective immunity against wild-type SARS-CoV-2 and the Delta variant: A prospective nationwide cohort study comparing three COVID-19 vaccination protocols in South Korea

**DOI:** 10.3389/fimmu.2022.968105

**Published:** 2022-09-23

**Authors:** Eliel Nham, Jae-Hoon Ko, Kyoung-Ho Song, Ju-Yeon Choi, Eu Suk Kim, Hye-Jin Kim, Byoungguk Kim, Hee-Young Lim, Kyung-Chang Kim, Hee-Chang Jang, Kyoung Hwa Lee, Young Goo Song, Yae Jee Baek, Jin Young Ahn, Jun Yong Choi, Yong Chan Kim, Yoon Soo Park, Won Suk Choi, Seongman Bae, Sung-Han Kim, Eun-Suk Kang, Hye Won Jeong, Shin-Woo Kim, Ki Tae Kwon, Sung Soon Kim, Kyong Ran Peck

**Affiliations:** ^1^ Division of Infectious Diseases, Department of Medicine, Samsung Medical Center, Sungkyunkwan University School of Medicine, Seoul, South Korea; ^2^ Department of Internal Medicine, Seoul National University Bundang Hospital, Seoul National University College of Medicine, Seongnam, South Korea; ^3^ National Institute of Infectious Diseases, Korea National Institute of Health, Korea Disease Control and Prevention Agency, Cheongju, South Korea; ^4^ Division of Infectious Diseases, Department of Internal Medicine, Gangnam Severance Hospital, Yonsei University College of Medicine, Seoul, South Korea; ^5^ Department of Internal Medicine, Severance Hospital, Yonsei University College of Medicine, Seoul, South Korea; ^6^ Division of Infectious Disease, Department of Internal Medicine, Yongin Severance Hospital, Yonsei University College of Medicine, Yongin, South Korea; ^7^ Division of Infectious Diseases, Department of Internal Medicine, Ansan Hospital, Korea University College of Medicine, Ansan, South Korea; ^8^ Department of Infectious Diseases, Asan Medical Center, University of Ulsan College of Medicine, Seoul, South Korea; ^9^ Department of Laboratory Medicine and Genetics, Samsung Medical Center, Sungkyunkwan University School of Medicine, Seoul, South Korea; ^10^ Department of Internal Medicine, Chungbuk National University College of Medicine, Cheongju, South Korea; ^11^ Department of Internal Medicine, School of Medicine, Kyungpook National University, Daegu, South Korea; ^12^ Division of Infectious Diseases, Department of Internal Medicine, Kyungpook National University Chilgok Hospital, School of Medicine, Kyungpook National University, Daegu, South Korea

**Keywords:** protective immunity, vaccination, neutralizing antibody, SARS-CoV-2, COVID-19

## Abstract

**Introduction:**

Despite vaccine development, the COVID-19 pandemic is ongoing due to immunity-escaping variants of concern (VOCs). Estimations of vaccine-induced protective immunity against VOCs are essential for setting proper COVID-19 vaccination policy.

**Methods:**

We performed plaque-reduction neutralizing tests (PRNTs) using sera from healthcare workers (HCWs) collected from baseline to six months after COVID-19 vaccination and from convalescent COVID-19 patients. The 20.2% of the mean PRNT titer of convalescent sera was used as 50% protective value, and the percentage of HCWs with protective immunity for each week (percent-week) was compared among vaccination groups. A correlation equation was deduced between a PRNT 50% neutralizing dose (ND_50_) against wild type (WT) SARS-CoV-2 and that of the Delta variant.

**Results:**

We conducted PRNTs on 1,287 serum samples from 297 HCWs (99 HCWs who received homologous ChAdOx1 vaccination (ChAd), 99 from HCWs who received homologous BNT162b2 (BNT), and 99 from HCWs who received heterologous ChAd followed by BNT (ChAd-BNT)). Using 365 serum samples from 116 convalescent COVID-19 patients, PRNT ND_50_ of 118.25 was derived as 50% protective value. The 6-month cumulative percentage of HCWs with protective immunity against WT SARS-CoV-2 was highest in the BNT group (2297.0 percent-week), followed by the ChAd-BNT (1576.8) and ChAd (1403.0) groups. In the inter-group comparison, protective percentage of the BNT group (median 96.0%, IQR 91.2–99.2%) was comparable to the ChAd-BNT group (median 85.4%, IQR 15.7–100%; *P* =0.117) and significantly higher than the ChAd group (median 60.1%, IQR 20.0–87.1%; *P <*0.001). When Delta PRNT was estimated using the correlation equation, protective immunity at the 6-month waning point was markedly decreased (28.3% for ChAd group, 52.5% for BNT, and 66.7% for ChAd-BNT).

**Conclusion:**

Decreased vaccine-induced protective immunity at the 6-month waning point and lesser response against the Delta variant may explain the Delta-dominated outbreak of late 2021. Follow-up studies for newly-emerging VOCs would also be needed.

## Introduction

Since its emergence in late 2019, coronavirus disease 2019 (COVID-19) has been a serious threat to humanity. Several vaccines against severe acute respiratory syndrome virus 2 (SARS-CoV-2) have been developed to overcome the ongoing pandemic, and both mRNA vaccines and adenovirus-vectored vaccines were approved in South Korea in 2021. Among them, BNT162b2, an mRNA vaccine developed by Pfizer and BioNTech (BNT), and AZD1222 ChAdOx1, an adenovirus-vectored vaccine developed by Oxford University and AstraZeneca (ChAd), were widely administered to the public (1, 2). Both vaccines were initially designed for administration in two doses at three- (BNT) or four- (ChAd) week intervals (3, 4), but vaccination strategies in South Korea have been amended several times because of serious vaccine-induced adverse effects and vaccine supplements (5–8). Meanwhile, breakthrough infections were observed earlier than expected, mostly due to the rapid spread of the Delta variant (B.1.617.2) (9), which reduces vaccine-induced neutralizing activity by three- to four-fold (9–17). A third dose of vaccine was introduced globally to overcome the Delta variant–predominant outbreak (18), while the newly emerging Omicron variant (B.1.1.529) with multiple mutations has become a following threat to vaccine-induced immunity.

To establish vaccination strategies for ongoing pandemic and continuously emerging SARS-CoV-2 variants, it is necessary to evaluate the vaccination strategies of the first year of COVID-19 vaccination and estimate the impact of Delta variant predominance during the 2021 outbreak. In particular, an ability to accurately estimate protective immunity based on the kinetics of neutralizing antibody titers is essential for predicting the persistence of the protective effect after a third vaccine dose (19–22). For this purpose, we investigated changes in the serologic response following vaccination using three major strategies implemented in South Korea: two doses of the BNT vaccine at a three-week interval (BNT group), two doses of the ChAd vaccine at a 12-week interval (ChAd group), and a single dose of ChAd followed by heterogeneous boosting with BNT at a 12-week interval (ChAd-BNT group) (8). For the estimation of protective immunity, we utilized the 20.2% of the mean PRNT titer of convalescent sera for 50% protective value as suggested by Khoury DS et al. (23).

## Methods

### Study population

This nationwide, multicenter, prospective cohort study was initiated under the leadership of the Korean Disease Control and Prevention Agency (KDCA) to evaluate the safety and efficacy of the national COVID-19 vaccination program. An earlier analysis of data from this study was published previously, which compared adverse effect and peak antibody response between the vaccination protocols (8). In the present analysis, we conducted a six-month follow-up analysis of the cohort. Healthcare workers (HCWs) from 10 hospitals in South Korea were recruited. To estimate protective immunity, we used 365 serum samples previously collected from reverse transcription polymerase chain reaction (RT-PCR)-confirmed COVID-19 patients who were infected in 2020 (24–27). Because the proportion of VOC among domestic cases was negligible before March 2021, those infected in 2020 are considered to have been non-VOC infections (28, 29). All participating HCWs and COVID-19 patients provided written informed consent, and the study protocol was approved by the institutional review board of each participating hospital.

HCWs receiving either the BNT or ChAd vaccines were recruited between March and April 2021. According to the early guidelines of the national vaccination program, BNT was assigned to HCWs designated for COVID-19 patient care, and ChAd was prescribed to those involved in non-COVID-19 patient care. The ChAd-BNT group was additionally recruited between May and June 2021. This cohort contained HCWs who experienced any adverse effects after the first dose of the ChAd vaccine in March 2021 and were willing to receive the BNT vaccine as a second dose. HCWs with a history of previous SARS-CoV-2 infection confirmed either by RT-PCR or detectable anti-nucleocapsid protein (NP) antibody at the baseline sampling were excluded from the present analysis.

### Data acquisition and sample collection

Data on the baseline characteristics of age, sex, height, body weight, and underlying diseases were collected. Use of acetaminophen (AAP) or non-steroidal anti-inflammatory drugs (NSAIDs) after vaccination was neither recommended nor prohibited. The reactogenicity data after the first and second vaccination were collected for seven days using an electronic diary (eDiary) format, which was developed based on phase III clinical trials of the vaccines (3–5). Side effects of pain, redness, swelling, fever, chill, myalgia, arthralgia, fatigue, headache, vomiting, and diarrhea were investigated, as was the need for AAP or NSAIDs to control side effects. Participants rated each symptom on a scale of 0 to 4 (0 for no symptoms, 1 for mild, 2 for moderate, 3 for severe, and 4 for critical). For AAP/NSAID use, a score of 0 was selected for no need for AAP/NSAIDs, 1 for 1–2 tablets per day, 2 for 3–4 tablets, 3 for 5–6 tablets, and 4 for more than 7 tablets per day (5).

Blood specimens were collected at five points, which varied by group: at week 0 (baseline for the ChAd and BNT groups), week 3 (after the first dose for the ChAd and BNT groups), week 5 (after the second dose for the BNT group), week 11 (before the second dose for the ChAd and ChAd-BNT groups), week 13 (the first waning point for the BNT group), week 14 (after the second dose for the ChAd and ChAd-BNT groups), and week 26 (the first waning point for the ChAd and ChAd-BNT groups and the second waning point for the BNT group).

### Laboratory procedures

#### Anti-SARS-CoV-2 spike protein total antibody assay

To estimate total antibody titers against the receptor binding domain (RBD) of the spike protein, the Elecsys^®^ Anti-SARS-CoV-2 S assay (Roche Diagnostics, Basel, Switzerland) was used. The kit was developed for *in vitro* qualitative and semi-quantitative measurement of anti-SARS-CoV-2 spike protein antibodies and uses an electro-chemiluminescence (ECLIA) method conducted with Cobas e modules (Roche Diagnostics). A recombinant RBD of the spike protein was used with the double-antigen sandwich principle. Although the antigen used in the kit is predominantly captured by IgG, IgA and IgM are also detectable (30). The range of measurement is 0.4–250 U/mL (up to 2,500 U/mL with onboard 1:10 dilution and up to 12,500 U/mL with onboard 1:50 dilution). Values higher than 0.8 U/mL were considered positive.

#### Anti-SARS-CoV-2 NP antibody assay

To detect anti-SARS-CoV-2 NP antibody induced by past SARS-CoV-2 infection, an Elecsys^®^ Anti-SARS-CoV-2 kit (Roche Diagnostics) was used. The double-antigen sandwich principle was used, and the ECLIA method was applied with Cobas e modules. The detectable isotypes included IgA and IgG, and a cut-off index greater than or equal to 1.0 was considered positive (31).

#### Plaque-reduction neutralization test (PRNT)

To evaluate the neutralizing activity of sera from vaccinated HCWs, PRNTs were conducted at the KDCA. PRNTs against WT SARS-CoV-2 were performed for 100 HCWs in each vaccination group. Briefly, 12-well plates were seeded with 2.5×10^5^ Vero cells (ATCC CCL-81)/mL/well and incubated at 37°C in a 5% CO_2_ incubator for 24 hours. Heat-inactivated (56°C for 30 minutes) serum samples in 96-well plates were serially diluted four-fold with Dulbecco’s Modified Eagles Medium containing 2% fetal bovine serum (FBS) and 1% penicillin/streptomycin. The diluted serum was incubated at 37°C in a 5% CO_2_ incubator for 1 hour. A dilution of 50 plaque forming unit/well of SARS-CoV-2 (βCoV/Korea/KCDC03/2020 NCCP No.43326) was prepared. Vero cells on a 12-well plate were inoculated with the serum and virus mixtures and incubated at 37°C and 5% CO_2_ for 1 hour. After the inoculums were removed, the cells were overlaid with 1 ml of Minimum Essential Medium containing 0.75% agarose and 2% FBS. The plates were incubated at 37°C and 5% CO_2_ for three days and then stained with 0.07% crystal violet, 10% formaldehyde, and 5% ethanol. The visualized plaques were counted. The 50% neutralizing dose (ND_50_) titer was calculated using Karber formula: log_10_ ND_50_ = m-Δ(∑p-0.5) (32). A 140 serum samples obtained after the second dose of vaccination (40 sera from the ChAd, 50 sera from the BNT, and 50 sera from the ChAd-BNT) were additional tested for both WT SARS-CoV-2 and the Delta variant (hCoV-19/Korea119861/KDCA/2021; NCCP43390). PRNTs of sera from convalescent COVID-19 patients were performed with the Vero E6 cell line (ATCC CRL-1586) using the same laboratory procedures.

#### Statistical analysis

Descriptive statics are presented as mean ± standard deviation (SD) or median {interquartile range (IQR)}. To compare baseline characteristics and clinical variables, one-way analysis of variance was used for continuous variables, and the chi-square test was used for categorical variables. For comparison of reactogenicity after the first and second doses, a summation of reactogenicity scores was used. To estimate the 50% protective neutralizing titer, 20.2% of the mean PRNT titer of convalescent sera was used based on a previous report that analyzed seven vaccine studies and one convalescent study (23). Convalescent sera from RT-PCR-confirmed COVID-19 patients collected between days 28 and 100 were used for this estimation (23, 33, 34). Subjects with an ND_50_ level equal to or higher than the estimated 50% protective neutralizing titer were considered to have protective immunity against SARS-CoV-2. For a quantitative comparison of protective immunity among the three vaccination strategies, the individual ND_50_ for each week after the first dose was calculated using the slope between sampling points. The percentage of HCWs with protective immunity during each week was compared among vaccination groups. For estimation of protective immunity against the Delta variant, we applied a conversion formula between the ND_50_ against WT SARS-CoV-2 and that against the Delta variant calculated from a linear regression model due to a limited number of samples used in PRNT against the delta variant. All *P* values were two-tailed, and values < 0.05 were considered to be statistically significant. All statistical analyses were performed using R version 4.1.1 (R Foundation for Statistical Computing, Vienna, Austria).

## Results

### Study population

**Figure 1:**
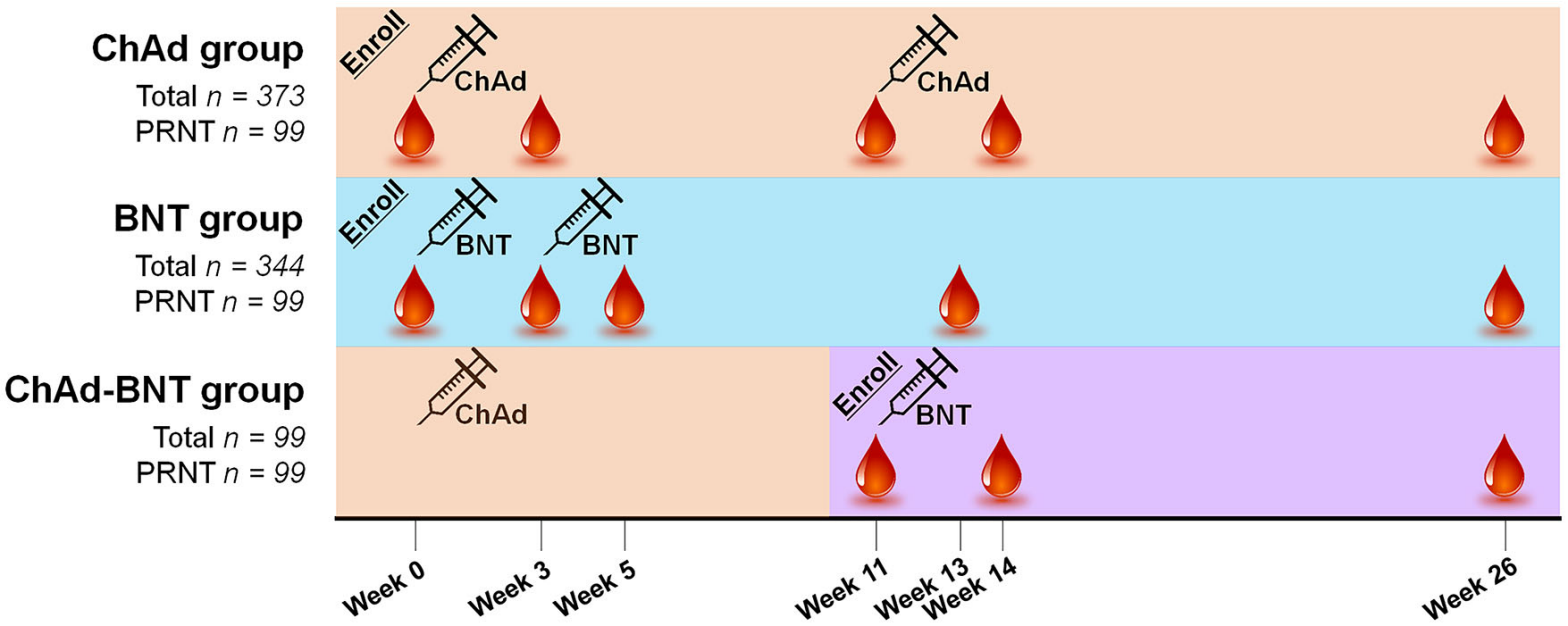
Vaccination schedule and sample acquisition timeline for each group. ChAd, AZD1222 ChAdOx1 vaccine; BNT, BNT162b2 vaccine; PRNT, plaque-reduction neutralizing test.

Overall, 822 HCWs were enrolled: 375 for the ChAd group, 347 for the BNT group, and 100 for the ChAd-BNT group. All the enrolled HCWs were tested for binding antibodies, and 100 subjects from each vaccination group were selected for PRNTs by order of enrollment. During follow-up, two HCWs in the ChAd group, three HCWs in the BNT group, and one HCW in the ChAd-BNT group dropped out, hence 816 HCWs were finally evaluated. The timeline for vaccination and blood sampling is illustrated in **Figure 1**. For the present analysis, the HCWs were followed for 26 weeks after their first vaccination. No cases of breakthrough infection occurred during the study period, which was identified by negative result of NP antibodies of the follow-up specimens. The baseline characteristics are presented in **Table 1**. The average age of the HCWs was 37.1 years, and the BNT group (average 35.3 years) was younger than the ChAd group (average 38.8 years). The subjects were mostly female (75.4%), and their average body mass index was 22.4 kg/m^2^. Only 12.6% of the HCWs had comorbidities, most of which were mild and well controlled.

**Table 1:** Baseline characteristics of the study participants.

Characteristics	Total participants (n = 816)	ChAd group		BNT group		ChAd-BNT group		*P* value	
		Total (n = 373)	PRNT (n = 99)	Total (n = 344)	PRNT (n = 99)	Total (n = 99)	PRNT (n = 99)	Total	PRNT
**Age, years**	37.1 ± 9.4	38.8 ± 9.4^*^	39.0 ± 10.0^*^	35.3 ± 9.3^*^	34.5 ± 8.7^*^	37.1 ± 9.2	37.1 ± 9.2	< 0.001	0.003
**Gender, female**	615 (75.4)	284 (76.1)	69 (69.7)	250 (72.7)	71 (71.7)	81 (81.8)	81 (81.8)	0.159	0.112
**BMI, kg/m^2^**	22.4 ± 3.0	22.4 ± 2.9	22.1 ± 2.5	22.4 ± 3.0	22.3 ± 3.3	22.0 ± 3.1	22.0 ± 3.1	0.416	0.727
**Comorbidity, any**	103 (12.6)	57 (15.3)	14 (14.1)	34 (9.9)	6 (6.1)	12 (12.6)	12 (12.1)	0.093	0.162
Hypertension	21 (2.6)	12 (3.2)	6 (6.1)	8 (2.3)	1 (1.0)	1 (1.0)	1 (1.0)	0.435	0.040
DM	11 (1.3)	7 (1.9)	1 (1.0)	3 (0.9)	1 (1.0)	1 (1.0)	1 (1.0)	0.483	1.000
Thyroid disease	21 (2.6)	11 (2.9)	2 (2.0)	8 (2.3)	0 (0.0)	2 (2.0)	2 (2.0)	0.813	0.363
Cardiovascular disease	5 (0.6)	2 (0.5)	1 (1.0)	2 (0.6)	0 (0.0)	1 (1.0)	1 (1.0)	0.862	0.604
Pulmonary disease	5 (0.6)	3 (0.8)	0 (0.0)	2 (0.6)	0 (0.0)	0 (0.0)	0 (0.0)	0.657	NA
Gastrointestinal disease	3 (0.4)	1 (0.3)	0 (0.0)	2 (0.6)	1 (1.0)	0 (0.0)	0 (0.0)	0.639	0.367
Liver disease	2 (0.2)	2 (0.5)	1 (1.0)	0 (0.0)	0 (0.0)	0 (0.0)	0 (0.0)	0.304	0.367
Renal disease	1 (0.1)	1 (0.3)	0 (0.0)	0 (0.0)	0 (0.0)	0 (0.0)	0 (0.0)	0.552	NA
Malignancy	8 (1.0)	4 (1.1)	1 (1.0)	2 (0.6)	1 (1.0)	2 (2.0)	2 (2.0)	0.428	0.776
Other	27 (3.3)	15 (4.0)	4 (4.0)	8 (2.3)	3 (3.0)	4 (4.0)	4 (4.0)	0.407	0.910
**Reactogenicity, score sum**									
After first dose	14.6 ± 17.8	20.1 ± 18.5^*^	23.1 ± 16.3^*^	7.6 ± 6.9^*^	7.4 ± 6.5^*^	39.9 ± 34.3^‡^	39.9 ± 34.3^‡^	< 0.001	< 0.001
After second dose	14.3 ± 15.5	7.5 ± 10.3^*†^	8.4 ± 9.5^*†^	19.8 ± 17.1^*^	22.7 ± 20.8^†^	20.2 ± 15.5^†^	20.2 ± 15.6^†^	< 0.001	< 0.001

Data are expressed as the number (%) of HCWs or mean ± SD.

^*^Statistically significant differences between ChAd and BNT groups. ^†^Statistically significant differences between ChAd and ChAd-BNT groups. ^‡^Reactogenicity data of ChAd-BNT group after the first dose of vaccination were available for 49 HCWs and have potential risk for recall bias. Reactogenicity score of ChAd-BNT group after first dose was significantly higher than ChAd and BNT groups.

ChAd, AZD1222 ChAdOx1 vaccine; BNT, Pfizer and BioNTech vaccine; PRNT, plaque-reduction neutralization test; BMI, body mass index; DM, diabetes mellitus; NA, not available.

To estimate protective immunity, we investigated 365 serum samples collected within 100 days of illness from 116 RT-PCR-confirmed patients (**Supplementary Figure 1**): 111 samples from 76 mild-to-moderate patients and 254 samples from 40 severe-to-critical patients. Fifty-two (44.8%) of the patients were male, and the average age was 44.2 years. According to PRNT ND_50_ titer, seroconversion occurred between the first and second weeks of illness, and the peak PRNT response was observed before day 21. One hundred forty-three serum samples collected after 28 days of illness were used as convalescent sera. The mean PRNT titer of the convalescent sera was 585.4 ± 854.6, and the median IQR was 206.8 (45.5–795.8). Patients with severe-to-critical illness (n = 70, median 599.2, IQR 199.5–1,681.0) showed higher titer than those with mild-to-moderate illness (n = 73, median 70.4, IQR 22.5–216.6; *P* < 0.001). Based on a previous estimation (23), we calculated a PRNT ND_50_ of 118.25 as the 50% protective neutralizing titer (20.2% of the mean).

### Measured neutralizing and anti-RBD antibody levels

**Figure 2:**
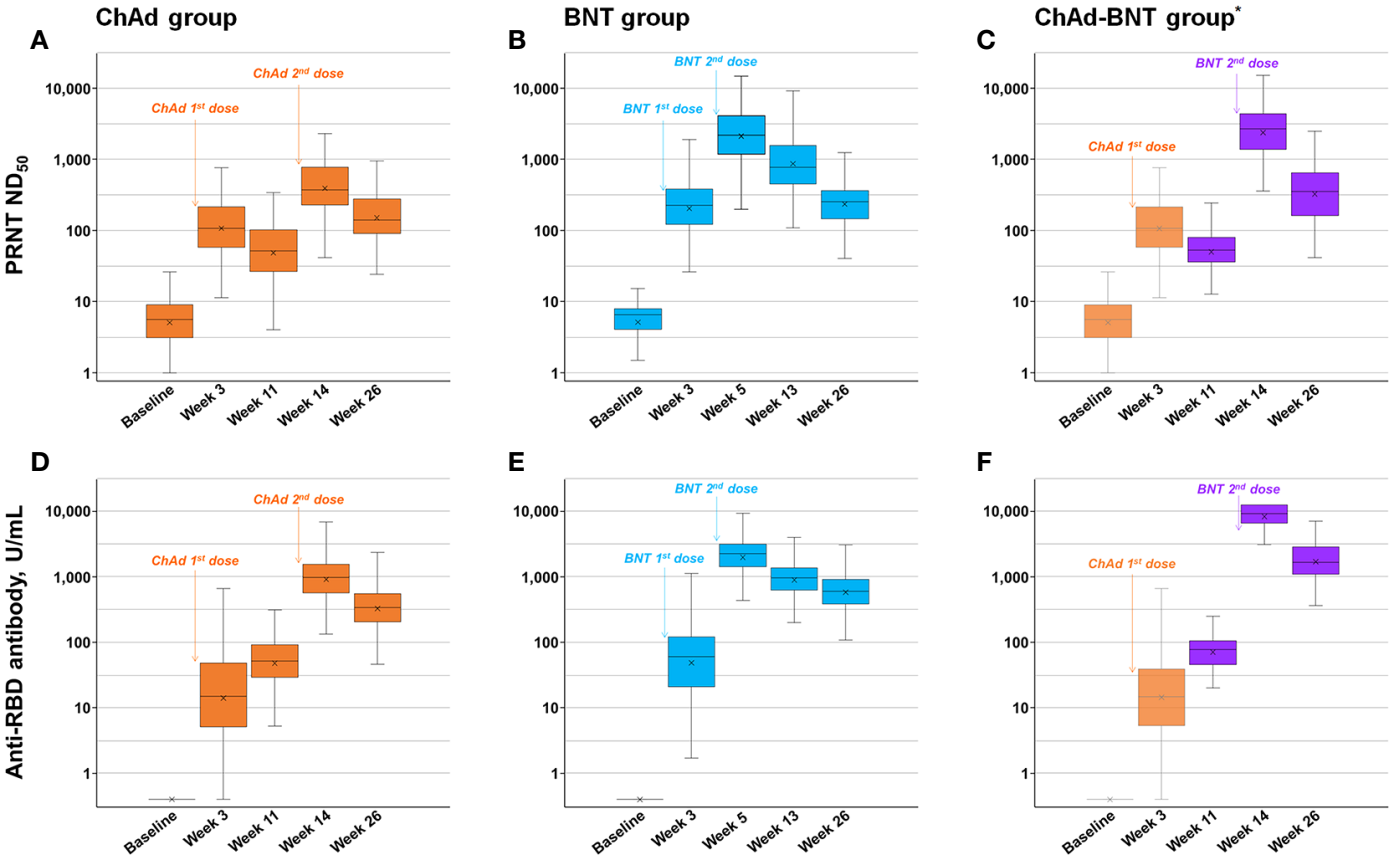
Measured neutralizing and anti-RBD antibody levels. Measured neutralizing antibody levels of ChAd **(A)**, BNT **(B)**, and ChAd-BNT **(C)** groups and anti-RBD antibody levels of ChAd **(D)**, BNT **(E)**, and ChAd-BNT **(F)** groups are depicted. ^*^Because the ChAd-BNT group was enrolled later, the baseline and week 3 antibody levels of the ChAd-BNT group were adopted from the ChAd group. ChAd, AZD1222 ChAdOx1 vaccine; BNT, BNT162b2 vaccine; PRNT, plaque-reduction neutralizing test; ND_50_, 50% neutralizing dose; RBD, receptor binding domain.

The measured binding and neutralizing antibody levels are presented in **Figure 2**. The PRNT ND_50_ titer peaked after the second dose in each vaccination group, as previously reported (8), and waned thereafter. The peak PRNT response of the ChAd-BNT group (median 2637.0, IQR 1377.0–4261.0) was comparable with that of the BNT group (median 2151.9, IQR 11.65.4–4030.5; *P* =0.375) and higher than that of the ChAd group (median 374.0, IQR 231.0–783.0; *P <*0.001). Six months (26 weeks) after the first dose, the ChAd-BNT group (median 355.1, IQR 160.9–649.0) showed a higher PRNT ND_50_ titer than both the ChAd (median 139.6, IQR 90.5–273.6; *P <*0.001) and BNT groups (median 253.6, IQR 145.8–365.7; *P* =0.003).

The peak anti-RBD antibody response of the ChAd-BNT group (median 9266.0, IQR 6590.8–12500.0) was significantly higher than that of the BNT group (median 2245.0, IQR 1431.8–3169.3; *P <*0.001) and ChAd group (median 972.0, IQR 567.0–1549.0; *P <*0.001). Six months after the first dose, the ChAd-BNT group (median 1663.0, IQR 1058.3–2769.0) showed a higher anti-RBD antibody titer than both the ChAd (median 341.0, IQR 202.8–555.5; *P <*0.001) and BNT groups (median 597.0, IQR 383.5–908.0; *P <*0.001).

### Estimated protective immunity against WT SARS-CoV-2 and Delta variant

**Figure 3:**
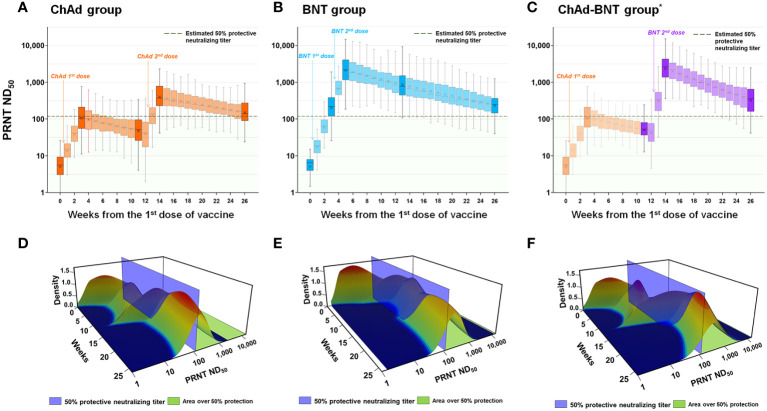
Estimation of protective immunity against WT SARS-CoV-2 for a 26-week period after vaccination. Individual PRNT ND_50_ values were calculated for each week after the first vaccine dose (transparent color) using the slope between the measured sampling points (dark color) and plotted for each vaccination group [ChAd **(A)**, BNT **(B)**, and ChAd-BNT **(C)**]. The distributions of PRNT ND_50_ titers for each week in ChAd **(D)**, BNT **(E)**, and ChAd-BNT **(F)** groups are presented using three-dimensional graphs. ^*^Because the ChAd-BNT group was enrolled later, the baseline and week 3 antibody levels of the ChAd-BNT group were adopted from the ChAd group. WT, wild-type; SARS-CoV-2, severe acute respiratory syndrome coronavirus 2; PRNT, plaque-reduction neutralizing test; ND_50_, 50% neutralizing dose; ChAd, AZD1222 ChAdOx1 vaccine; BNT, BNT162b2 vaccine.

For a quantitative comparison of the estimated protective immunity against WT SARS-CoV-2 among the vaccination groups, the percentage of HCWs with protective immunity (PRNT ND_50_ ≥118.25, **Figure 3**) during each week was estimated. At each measured time point, 0.0%, 45.5%, 16.2%, 92.9%, and 58.6% of the HCWs in the ChAd group; 0.0%, 77.8%, 99.0%, 98.0%, and 84.8% of the HCWs in the BNT group; and 0.0%, 45.5%, 9.1%, 99.0%, and 83.8% of the HCWs in the ChAd-BNT group had protective immunity. The 26-week cumulative percentage of HCWs with protective immunity was highest in the BNT group (2297.0 percent-week), followed by the ChAd-BNT (1576.8) and ChAd groups (1403.0). When the median values were compared among groups, the BNT group (median 96.0%, IQR 91.2–99.2%) was comparable to the ChAd-BNT group (median 85.4%, IQR 15.7–100%; *P* =0.117), and they were both significantly higher than the ChAd group (median 60.1%, IQR 20.0–87.1%; *P <*0.001).

**Figure 4:**
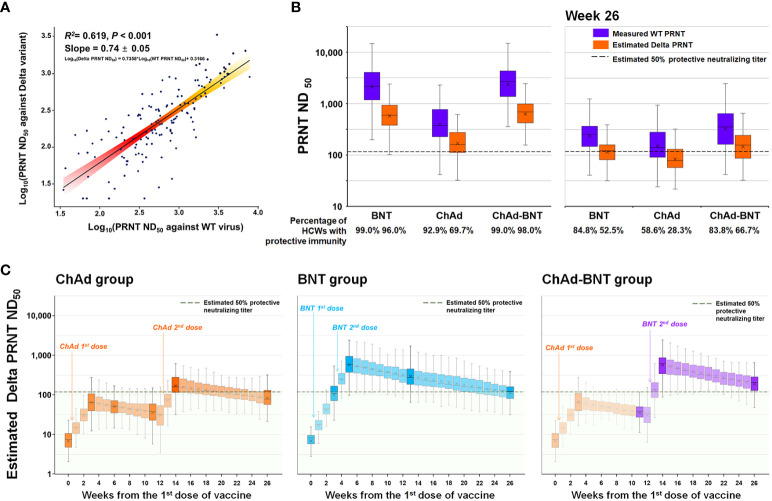
Estimated protective immunity against the Delta variant using the PRNT correlation equation. A correlation equation between the PRNT ND_50_ against WT SARS-CoV-2 and that against the Delta variant was deduced using 140 serum samples that underwent PRNT against the two strains simultaneously **(A)**. The PRNT ND_50_ against the Delta variant was calculated for each measured time point **(B)**, and the PRNT ND_50_ for each week was estimated using the slope between measured points **(C)**. WT, wild-type; SARS-CoV-2, severe acute respiratory syndrome coronavirus 2; PRNT, plaque-reduction neutralizing test; ND_50_, 50% neutralizing dose; ChAd, AZD1222 ChAdOx1 vaccine; BNT, BNT162b2 vaccine.

Using the test results from 140 serum samples that underwent simultaneous PRNTs against WT SARS-CoV-2 and the Delta variant, we deduced a correlation equation: Log_10_(Delta PRNT ND_50_) = 0.7358*Log_10_(WT PRNT ND_50_) + 0.3166 (**Figure 4A**). There was no statistical difference between the measured and calculated values at each sampling points (**Supplementary Table 1**). Using that equation, the WT PRNT ND_50_ at each measured time point was converted to the Delta PRNT ND_50_ (**Figure 4B**). At each measured time point, 0.0%, 20.2%, 2.0%, 69.7%, and 28.3% of the HCWs in the ChAd group; 0.0%, 46.5%, 96.0%, 91.9%, and 52.5% of the HCWs in the BNT group; and 0.0%, 20.2%, 3.0%, 98.0%, and 66.7% of the HCWs in the ChAd-BNT group were estimated to have protective immunity against the Delta variant. The 26-week cumulative percentage of HCWs with protective immunity against the Delta variant was highest in the BNT group (1973.7 percent-week), followed by the ChAd-BNT (1318.2) and ChAd groups (737.4) (**Figure 4C**). When the median values were compared among groups, the BNT group (median 89.9%, IQR 59.6–95.2%) was comparable to the ChAd-BNT group (median 66.2%, IQR 4.8–98.0%; *P* =0.280), and they were both significantly higher than the ChAd group (median 24.2%, IQR 7.3–49.7%; *P <*0.001). For the validation of estimated Delta PRNT ND_50_, calculated values were compared with measured values of Delta PRNT ND_50_ using identical sera. The peak response and 6-months waning point were compared, and no statistically significant differences were noticed between estimated and measured Delta PRNT ND_50_ (all *P* > 0.05; **Supplementary Figure 2**).

## Discussion

Despite the rapid development and wide distribution of SARS-CoV-2 vaccines, the COVID-19 pandemic is ongoing after more than two years, mainly because emerging VOCs escape vaccine-induced immunity (8, 10, 12, 13, 15, 29). *In vitro* studies showed that the vaccine-induced neutralizing activity against the Delta variant would decrease by 3–10-fold compared with that against the WT virus (8, 10, 13, 15), but the decrease in vaccine-induced protective immunity over time has not been well evaluated. In this analysis, the PRNT titers against the WT and Delta variant showed a linear correlation with a logarithmic scale, which allowed us to deduce a correlation equation of log_10_ PRNT ND_50_ against the WT and Delta variant. Using the previously reported estimation method for a 50% protective neutralizing titer (23), we calculated the percentage of HCWs with protective immunity against the WT and Delta variant. The ChAd group showed the lowest WT PRNT titer (8), and the percentage of HCWs in the ChAd group with protective immunity against the WT at the 6-month waning point decreased to 58.6%, whereas more than 80% of the HCWs in the BNT and ChAd-BNT groups maintained their immunity. According to the calculated Delta PRNT titers, the percentage of HCWs with protective immunity decreased to 28.3%, 52.5%, and 66.7% in the ChAd, BNT, and ChAd-BNT groups, respectively. According to a KDCA report, the nationwide overall protection effect of two vaccine doses, regardless of vaccination protocol, was 58.2% during the fifth week of December 2021, the peak of the Delta-predominant outbreak (35). The protective effect of each vaccination protocol has not been precisely calculated, but practitioners frequently reported breakthrough infections in the ChAd group (36). Although accurate validation of our estimation is not possible due to lack of an individual vaccination and SARS-CoV-2 infection database, our results regarding the estimated waning of protective immunity appear to reflect the real-world outbreak situation.

Of note, the cumulative protective percentage for the 6-month estimation was highest in the BNT group (2297.0 percent-week), followed by the ChAd-BNT (1576.8) and ChAd groups (1403.0). The peak PRNT response was achieved first by the BNT group, whereas the titer was highest in the ChAd-BNT group. Other published reports also suggest that heterologous boosting induced higher immunologic responses (37–40), and we further evaluated peak and waning response of antibodies according to the timeline to estimate cumulative protective effect. When the COVID-19 vaccine was introduced, the optimal interval between the first and second vaccine doses was debatable (20, 41). Our estimation suggests that reaching the peak PRNT titer sooner would provide better protective immunity for a 6-month period. However, the actual outbreak phenomenon was more complicated because the Delta-dominant large scale outbreak started 3–6 months after the initiation of wide-scale vaccination. The South Korean healthcare authority implemented a third dose as a booster shot to overcome the Delta-dominated outbreak (35), and that third dose was reported to provide a higher PRNT titer against VOCs, including the Omicron variant (42). Follow-up studies estimating the protective immunity for emerging VOCs need to continue.

Although many studies have reported the vaccine-induced immune response (9, 16, 17, 43–48), few performed the serial neutralizing test because it requires a biosafety level 3 facility, experienced personnel, and enormous time. We could not perform Delta PRNTs for enough serum samples to present the kinetics with measured Delta PRNT titers. Instead, we noticed a linear correlation between the log_10_ PRNT ND_50_ against the WT and Delta variant and deduced a correlation equation between them. With the calculated Delta PRNT value, we could estimate protective immunity against the Delta variant. However, estimating protective immunity against the Omicron variant would require additional validation. After the third vaccination, a 6–17-fold reduction of PRNT titer against the Omicron variant (compared with the WT) was observed and the correlation between PRNT ND_50_ values against WT and Omicron variant was poor (49). Interpretation of immunoassays measuring binding antibodies needs to be cautious because most assays are produced based on the receptor binding domain of WT SARS-CoV-2. Continuous efforts are needed to establish ways to measure meaningful antibody titers that predict protective immunity, and our data could provide background knowledge for such efforts using the standard vaccination dose.

The present study has several limitations. First, as an observational cohort study, the study population had differences in demographics and underlying diseases. Specifically, the HCWs in the BNT group were younger than those in the other groups. Second, because of the different intervals between the first and second doses with different vaccines, the follow-up period after the second vaccination differed among the groups. Third, we did not include cell-mediated immunity in the present analysis. However, the present study focused on protective immunity in terms of neutralizing antibody titers in PRNTs, a gold standard test method for measuring neutralizing activity. Fourth, as the estimation of protective immunity in the present study is based from the pooled analysis of various vaccine studies and a correlation equation between WT and Delta PRNT (23), it would not exactly reflect protective immunity of each individual. An elaborate calculation of protective immunity against VOCs would be extremely challenging due to rapidly changing outbreak situations. Despite these limitations, we could compare each vaccine protocols with regard to the actual outbreak situation by utilizing the estimated values of protective immunity.

In conclusion, the percentage of HCWs with protective immunity against WT SARS-CoV-2 at the 6-month waning point maintained over 80% in the BNT and ChAd-BNT groups but only 58.6% in the ChAd group. Further decreases in protective immunity against the Delta variant may explain the Delta-predominated outbreak of late 2021. Follow-up studies need to be conducted for newly emerging VOCs.

## Data availability statement

The raw data supporting the conclusions of this article will be made available by the authors, without undue reservation.

## Ethics statement

The studies involving human participants were reviewed and approved by Samsung Medical Center and all other included institutions. The patients/participants provided their written informed consent to participate in this study.

## Author contributions

Conceptualization: EN, J-HK, K-HS, HJ, S-WK, KK, SK, and KP. Investigation: EN, J-HK, K-HS, EK, KL, YS, YB, JA, JC, YK, YP, WC, SB, S-HK, E-SK, HJ, S-WK, and KK. Laboratory work: J-YC, H-JK, BK, H-YL, K-CK, H-CJ, and SK. Data analysis: EN and J-HK. Writing – original draft: EN and J-HK. Writing – review and editing: HJ, S-WK, KK, SK and KP. All authors contributed to the article and approved the submitted version.

## Funding

This work was supported by a Research Program funded by the Korean Disease Control and Prevention Agency (#2020-ER5328-00 and #2021-ER2601-00), a Samsung Medical Center Grant (#SMO1210321), and a SNUBH Research Fund (#14-2021-023).

## Acknowledgments

We would like to thank Jinseob Kim (Zarathu Co., Ltd.) for advice on statistics and figure development.

## Conflict of interest

The authors declare that the research was conducted in the absence of any commercial or financial relationships that could be construed as a potential conflict of interest.

## Publisher’s note

All claims expressed in this article are solely those of the authors and do not necessarily represent those of their affiliated organizations, or those of the publisher, the editors and the reviewers. Any product that may be evaluated in this article, or claim that may be made by its manufacturer, is not guaranteed or endorsed by the publisher.
