# Supplementary material for: Kinetics of vaccine-induced neutralizing antibody titers and estimated protective immunity against wild-type SARS-CoV-2 and the Delta variant: A prospective nationwide cohort study comparing three COVID-19 vaccination protocols in South Korea
Source: Front Immunol. 2022 Sep 23;13:968105. doi: 10.3389/fimmu.2022.968105 (PMC9538478; doi:10.3389/fimmu.2022.968105)
Supplement: Supplementary file 1 [file DataSheet_1.docx]

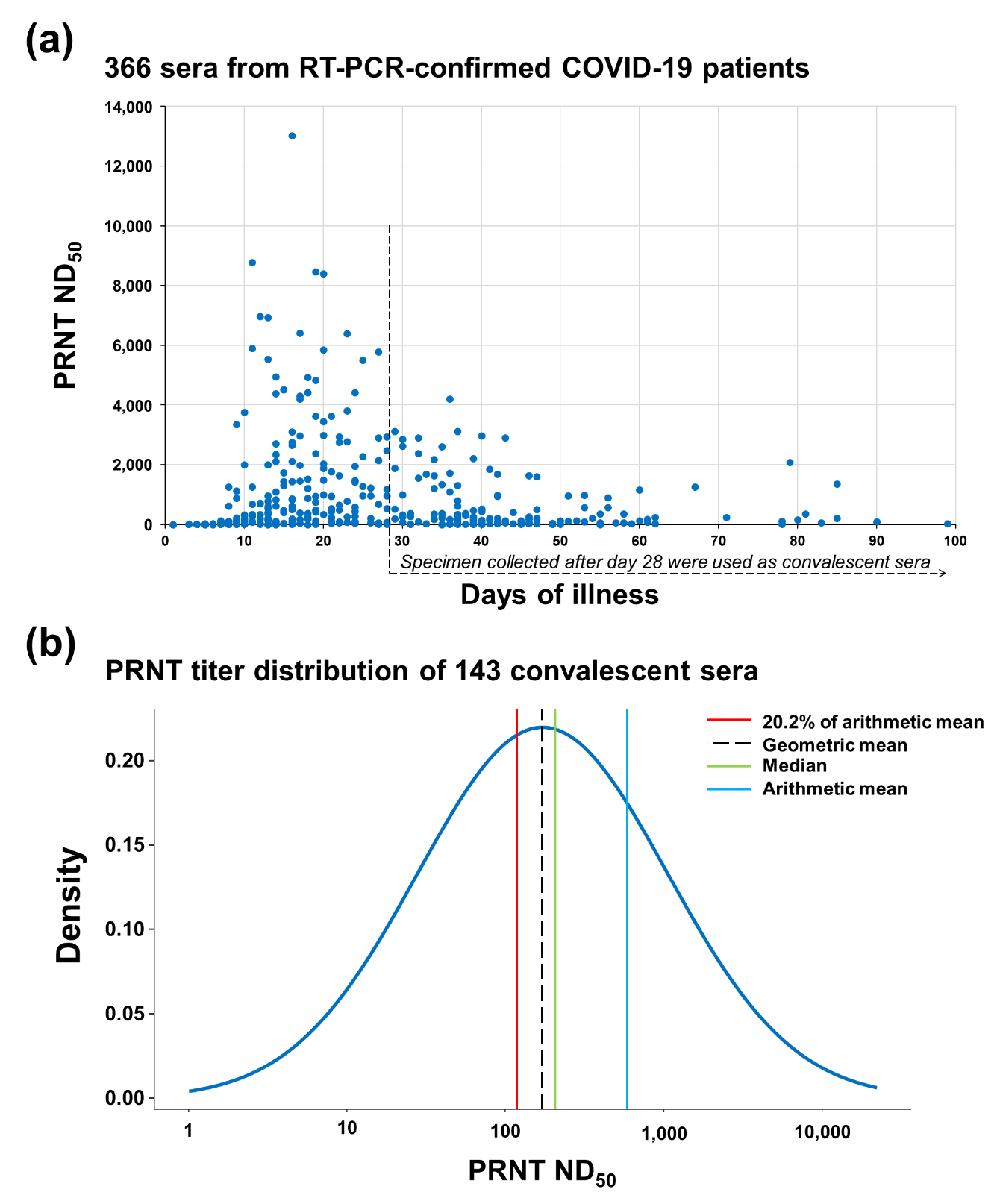


**Supplementary Figure 1. Estimation of 50% protective immunity using sera from COVID-19 patients**

(a) Time-dependent plot of PRNT ND_50_ in 365 serum samples from 116 RT-PCR-confirmed COVID-19 patients. The peak PRNT response was observed before day 21 of illness, and convalescent serum was defined as that collected after 28 days of illness. (b) The PRNT ND_50_ distribution in 143 convalescent serum samples. A PRNT ND_50_ of 118.25 was estimated to represent a 50% protective neutralizing titer (20.2% of the mean).

Abbreviations: RT-PCR, real-time polymerase chain reaction; COVID-19, coronavirus disease 2019; PRNT, plaque-reduction neutralizing test; ND_50_, 50% neutralizing dose


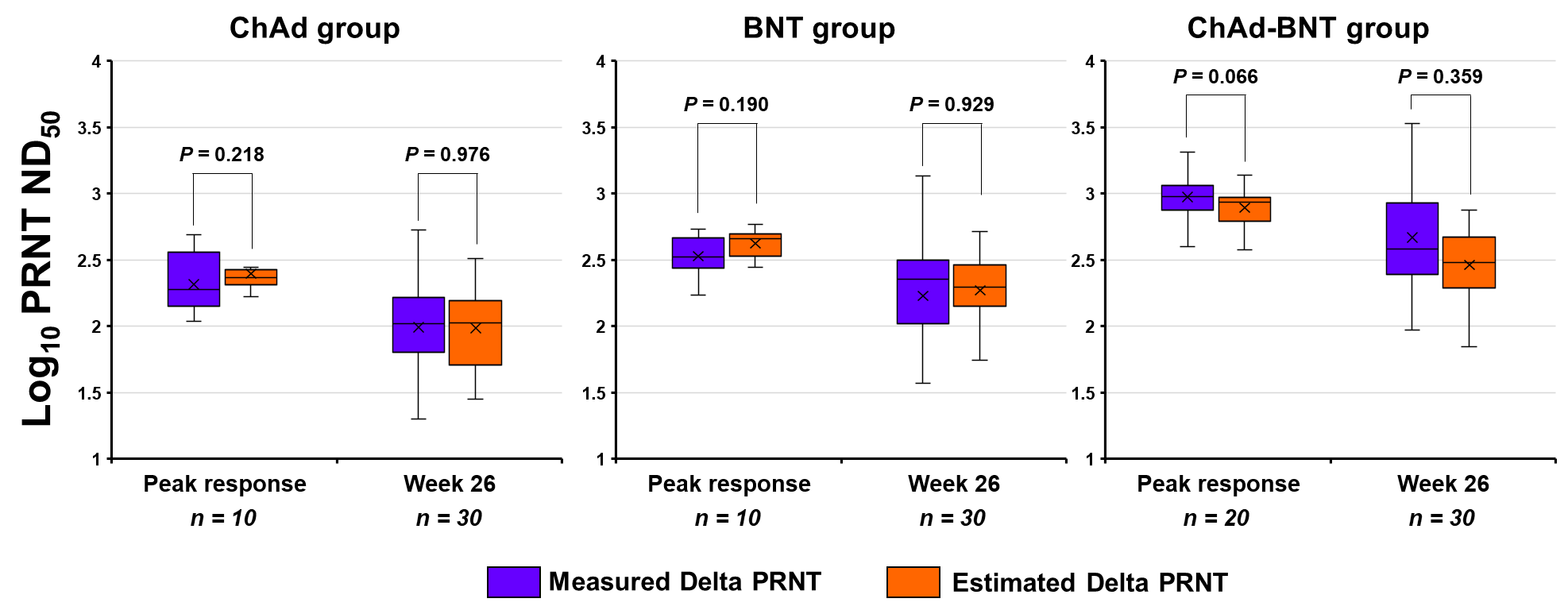


**Supplementary Figure 2. Comparison of estimated and measured Delta PRNT**

For the validation of estimated Delta PRNT ND_50_, calculated values were compared with measured values of Delta PRNT ND_50_ using identical sera. The peak response and 6-months waning point were compared, and no statistically significant differences were noticed between estimated and measured Delta PRNT ND_50_.

Abbreviations: PRNT, plaque-reduction neutralizing test; ND_50_, 50% neutralizing dose; ChAd, AZD1222 ChAdOx1 vaccine; BNT, BNT162b2 vaccine

**Supplementary Table 1. Comparison between the measured Delta PRNT and calculated Delta PRNT titers, according to the sampling points**

| **Sampling point** | **Log_10_ Delta PRNT titer, measured value** | **Log_10_ Delta PRNT titer, calculated value** | ***P* value** |
| --- | --- | --- | --- |
| 3^rd^ sampling, *n = 10* | 2.5 (2.5–2.7) | 2.7 (2.6–2.7) | 0.102 |
| 4^th^ sampling, *n = 40* | 2.7 (2.2–3.0) | 2.7 (2.3–2.9) | 0.902 |
| 5^th^ sampling, *n = 90* | 2.3 (2.0–2.5) | 2.3 (2.0–2.5) | 0.961 |

Data are expressed as median (IQR).

Abbreviations: PRNT, plaque-reduction neutralizing test
